# Secondary Aortoduodenal Fistula Presenting as Gastrointestinal Bleeding and Fungemia

**DOI:** 10.7759/cureus.5575

**Published:** 2019-09-05

**Authors:** Radhakrishna Vegunta, Rathnamitreyee Vegunta, Gurusaravanan Kutti Sridharan

**Affiliations:** 1 Oncology, Sanford Health/University of North Dakota School of Medicine and Health Sciences, Fargo, USA; 2 Internal Medicine, Westchester Medical Center, New York, USA; 3 Internal Medicine, The University of Arizona, Tucson, USA

**Keywords:** aortoenteric fistula, gi bleed, candida, fungemia, revascularisation, aortic graft, polymicrobial, sepsis

## Abstract

A 55‐year‐old African American man with a history of abdominal aortic pseudoaneurysm repair presented to the ED with complaints of black-colored stools mixed with fresh blood and fever for three days duration. The exam was unremarkable except for abdominal bruits and pallor. CT angiogram showed perigraft fluid collection, bowel wall thickening, and loss of normal fat planes between the aorta and adjacent bowel at the level of the third portion of the duodenum. Polymicrobial infection was noted in the aortic graft and blood cultures grew Candida. The patient underwent urgent removal of the infected graft, duodenal repair along with appropriate antimicrobial therapy. He did well postoperatively and was discharged in a stable condition. Our case highlights the importance of maintaining a high index of suspicion of aortoenteric fistula (AEF) when a patient with a prior abdominal aortic graft develops gastrointestinal (GI) bleeding as this condition is universally fatal if unrecognized.

## Introduction

An aortoenteric fistula (AEF) is an abnormal communication between the aorta and an adjacent loop of the bowel. AEFs are uncommon life-threatening conditions. AEF can be classified as primary or secondary based on their etiology. Primary AEFs are uncommon and occur when a previously untreated aneurysm erodes into the adjacent bowel. Secondary AEF commonly follows a surgically placed aortic graft but can occur following any aortic reconstruction including endovascular aneurysm stent-graft repair or the use of bare metal aortic stents [1-2]. We report a rare case of secondary aortoduodenal fistula following abdominal aortic aneurysm repair with aortobiiliac reconstruction presenting with a gastrointestinal (GI) bleed and complicated by fungemia.

## Case presentation

**Figure 1:**
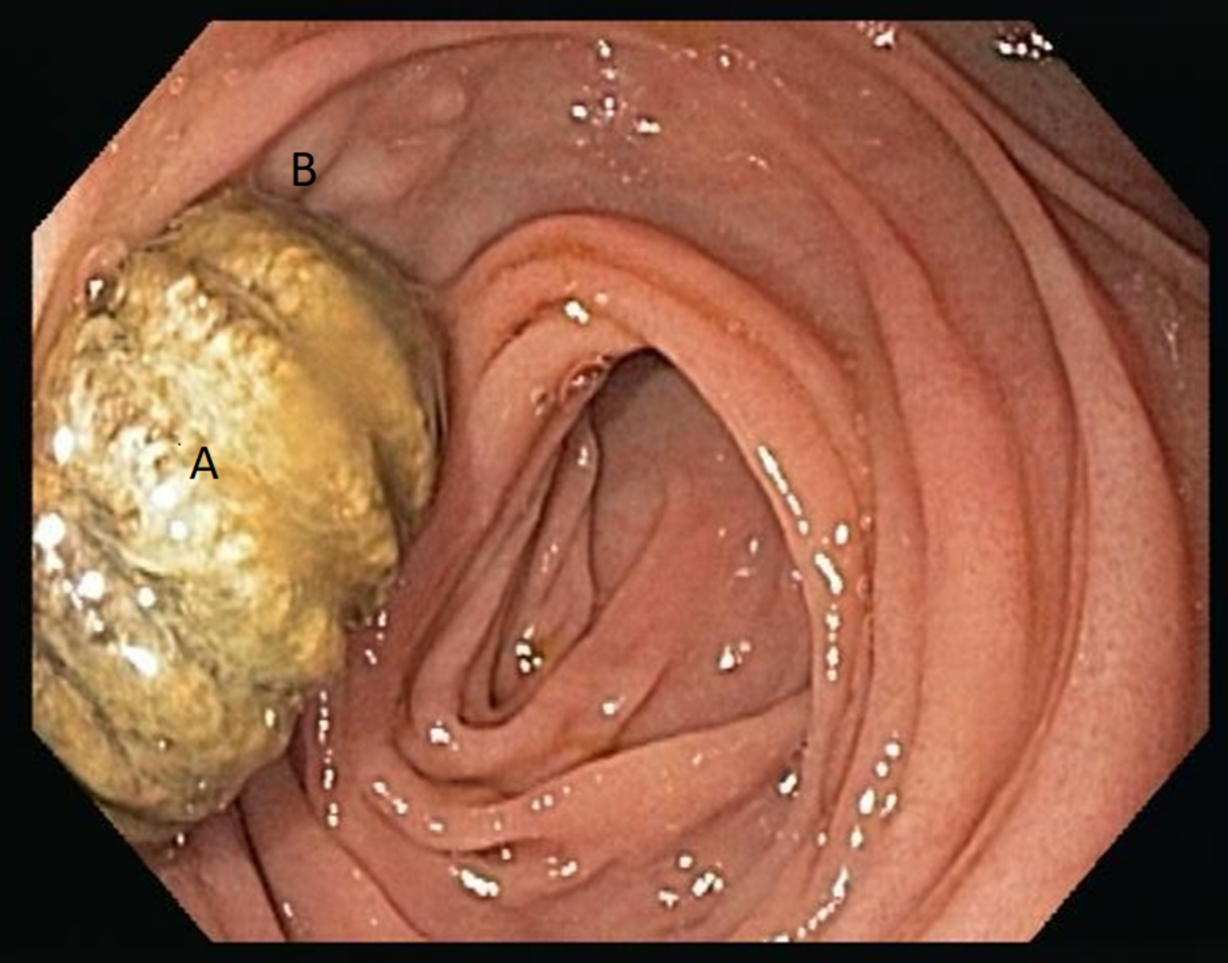
Upper endoscopy images showing aortic graft showing aortic graft material (A) eroding into the third part of the duodenum (B).

A 55‐year‐old African American man with a history of abdominal aortic pseudoaneurysm repair with aortobiiliac reconstruction was admitted with a chief complaint of dark‐colored stools, bright red blood in rectum, and fever for the past three days. His past medical history was significant for diabetes mellitus, hypertension, peripheral vascular disease, and cholelithiasis. He had a history of small bowel obstruction due to adhesions that was treated surgically. On exam, he appeared to be in mild distress. His vitals were stable, the abdomen was soft, nontender with normal bowel sounds with midline abdominal bruit. His labs revealed low hemoglobin level and elevated leukocyte count. Upper endoscopy showed graft material eroding into the third portion of the duodenum most likely due to fistulization from the aorta (Figure 1). 

The CT angiogram showed perigraft fluid collection, bowel wall thickening, and loss of normal fat planes between the aorta and adjacent bowel at the level of the third portion of the duodenum. The patient underwent removal of the infected aortobiiliac graft and repair of the duodenum. Blood cultures grew *Candida albicans*, and graft material cultures grew *Escherichia coli*, *C. albicans*, and anaerobes. He was treated with appropriate antimicrobials including fluconazole. He had a prolonged hospitalization but recovered well and was discharged in a stable condition. This case has been presented before (Abstract: Radha Krishna Vegunta, Aortoenteric Fistula: A Rare Cause of Gastrointestinal Bleeding. Hospital Medicine; May 17, 2013).

## Discussion

The incidence of secondary AEF is 0.3%-1.6% [3]. Aortoduodenal fistulae are the most common AEFs [4]. The most common site affected is the third and fourth parts of the duodenum. The most frequent presenting feature is upper GI bleeding, which can range from a minor “herald” bleed to exsanguinating hemorrhage. Occasionally, AEF may manifest with atypical, nonspecific symptoms such as fever, sepsis, or unexplained abdominal pain. The pathogenesis of AEF has not been fully elucidated but it has been postulated that a combination of chronic low-grade infection of the aortic graft and repetitive pressure on the intestine from aortic pulsations leads to the formation of these fistulas [5]. CT scan is the first-line diagnostic modality for evaluation of suspected AEF, followed by endoscopy and arteriography. There are many mimics of AEFs including perigraft infection without fistulization, retroperitoneal fibrosis, infected aortic aneurysm, and infectious aortitis. The management goal of AEF is to control hemorrhage and treat infection and maintain adequate distal perfusion. Aggressive fluid resuscitation and blood transfusions should be guided by invasive monitoring. Diagnostic tests and surgical evaluation need to be done expeditiously. Traditional treatment of AEF is graft excision and establishing an extra-anatomic bypass. Alternatives to this are in situ graft replacement and simple graft excision alone. Endovascular repair is a less invasive alternative therapeutic option, particularly for the rapid control of bleeding and may serve as a bridging therapy to open repair, if they have sepsis or other infectious complications. Broad-spectrum antibiotic therapy should be initiated immediately after the diagnosis is established with coverage for Gram-positive, Gram-negative, and anaerobes and continued postoperatively based on culture reports. Fungal infections of the vascular graft infection are rare [6]. However, it could be more common than previously thought secondary to advancements in medical science which have enabled immunocompromised patients to be candidates for risky vascular procedures thereby increasing the chance of opportunistic fungal infections. Candida was one of the two most common organisms found in aortic graft infections in two large retrospective series of AEF [7-8]. In view of the above findings, it may be reasonable to add empiric antifungal therapy in patients suspected of having aortoduodenal fistula. Our patient had no risk factors for fungal infection except for uncontrolled diabetes mellitus and Candida was not isolated from any other source making aortobiiliac graft infection the likely source of candidemia. During our search, we did not find any case report of secondary AEF presenting with GI bleed and candidemia due to primary fungal infection of aortobiiliac graft and this makes our case unique. The presence of Candida and polymicrobial bacteria in blood in a patient presenting with GI bleeding may suggest an aortoduodenal fistula and help in diagnosing this life-threatening condition [9].

## Conclusions

Aortoenteric fistula is a life-threatening condition associated with high morbidity and mortality and it can also pose a diagnostic dilemma. There are many mimics of AEFs including perigraft infection without fistulization, retroperitoneal fibrosis, infected aortic aneurysm, and infectious aortitis. AEF should always be on the differential in patients presenting with GI bleeding who have a history of abdominal aortic aneurysm repair. Also, aortoduodenal fistula should be strongly suspected in any patient with a history of aorta repair presenting with GI bleeding and candidemia. Prompt diagnosis and early surgical intervention are required to prevent a lethal outcome.
